# Dysphagia in Tuberculous Laryngitis

**Published:** 1908-02-22

**Authors:** George A. Crace-Calvert

**Affiliations:** Vale of Clwyd Sanatorium, North Wales


### Editor's Letter-Box.

### DYSPHAGIA IN TUBERCULOUS LARYNGITIS.

Sir,—I have read with interest the articles on laryngology and rhinology which have appeared in The Hospital of late. In particular the last one, on "The Dysphagia of Tuberculous Laryngitis," has interested me, as I have at present under treatment a case of tuberculous laryngitis where painful deglutition is a marked feature. There is a large amount of infiltration of the epiglottis and both arytenoids, with a large inter-arytenoid ulcer and superficial ulceration of the epiglottis and arytenoids. There is also much secretion of thick yellowish mucus.

I have tried spraying with a 5 per cent. solution of cocaine in oil, but, possibly owing to the mucus, it has not had much effect. I am at present using a mixture of orthoform and gum acacia—equal parts—as an insufflation, and it acts splendidly; but the point I wish to call attention to is that the anæsthetic effect begins in a few minutes and passes off completely in less than fifteen minutes. This no doubt is an anomaly, but may be worth recording, as it is so very different from the times stated in the article—namely, nearly an hour to fully develop, and lasting often for twelve hours.

Yours faithfully,

George A. Crace-Calvert, M.B.

Vale of Clwyd Sanatorium, North Wales,
February 18, 1908.

